# A Suggested New Term and Definition to Describe the Cumulative Physiological and Functional Effects of Non-injurious Head Impacts

**DOI:** 10.3389/fneur.2022.799884

**Published:** 2022-03-31

**Authors:** Andrew P. Lavender, Julia Georgieva, Ryusuke Takechi

**Affiliations:** ^1^School of Science, Psychology and Sport, Federation University Australia, Ballarat, VIC, Australia; ^2^Curtin School of Allied Health, Curtin University, Perth, WA, Australia; ^3^Curtin Health Innovation Research Institute, Curtin University, Perth, WA, Australia; ^4^Curtin School of Population Health, Curtin University, Perth, WA, Australia

**Keywords:** subconcussion, concussion, head impact, mild traumatic brain injury, blood brain barrier

## Introduction

In recent decades, there has been an increase in research on sport-associated concussion as a result of neurological symptoms experienced by high-profile and retired athletes, consequently lawsuits with large media coverage have ensued. Studies have linked repeated incidence of concussive events with some neurological conditions such as chronic traumatic encephalopathy (CTE) (1). This profound link clearly indicates the importance for researchers into the acute and chronic effects of concussion to make participation in sport safer for both professional and amateur/community athletes during their playing careers and beyond.

While this line of research in concussion is both interesting and important, research investigating the occurrence of very minor head impacts that occur repeatedly are relatively few. These impacts are performed deliberately in the form of an important skill, or part thereof, of the sport and do not result in concussion or any detectible injury. These repeated head impacts include those experienced by soccer players when heading the ball, rugby players when tackling and boxers in sparring practice and competitive bouts. While the effects on brain health among people who experience such minor repeated head impacts have not been well-researched in the past, this area of research has gained momentum in recent years and the term “subconcussion” has been used to describe the anatomical, physiological and cognitive effects of light head impacts in humans and rodent models (2–5). This term and definition for subconcussion is distinct from the phenomenon we are investigating in our research and this article is intended to draw the distinction between these two phenomena and explain the importance of using a new term to add clarity to research in brain health regarding mild head impacts.

## Subconcussion

Subconcussion has been defined as “a cranial impact that does not result in a known or diagnosed concussion on clinical grounds” (6). Hwang et al. (7) defined subconcussion as “a head impact that does not result in clinically observable deficits … resulting from low levels of head impact that have the potential to cause significant long-term neurological damage.” Another group has provided a description of the phenomenon calling it “repetitive subconcussive head impacts (RSHI)” (8). This definition adds the important aspect of repeated events of the kind experienced by sport participants. Longitudinal changes in a measure of eye function called near point convergence (NPC) has been shown in a study of American Football players across a football season. The authors concluded that the altered NPC was due to subconcussive impacts across the season which should be considered in a new definition (9).

Typically, it is believed that movement of the brain within the protective skull may result in subconcussion. However, it is interesting to note that such brain movement may occur in the absence of a blow to the head. A recent study induced rapid head movements in volunteers by asking them to visually track a fast-moving soccer ball. The volunteers were also required to perform a series of rapid head movements in coronal, sagittal and horizontal planes and a series of cognitive and neurological function tests, including the Balance Error Scoring System, King-Devick assessment, and the Standard Assessment of Concussion were performed. The investigation found no neurological deficits, although the authors reported a strong trend toward reduced BESS scores, which is in line with previous similar research (10). This study also performed some modeling of the brain movement within the skull and found it was very low in these voluntary rapid movements.

Another phenomenon that has gained attention in recent years is effect of low level blast impacts producing signs and symptoms associated with subconcussion. A recent investigation addressed the acute effects of subconcussive blast exposure in military personnel by assessing cognitive function in soldiers after such events. The authors reported that, in the Automated Neuropsychological Assessment Metrics 4 TBI-MIL (ANAM), those exposed to blasts exhibited statistically significant lower scores than the unexposed control group (11). Military personal called “Breachers” are often exposed to low level blasts during performance of their duties and in training exercises. An early study of this phenomenon assessed Serum samples, neurocognitive performance, and self-reported symptoms in twenty one defense force personnel before, during and following a 2-week training course. Significant declines in performance scores such as reaction time and as well as composite biomarker scores were apparent in some individuals. The authors also reported a correlation between these reduced performance and biomarker scores and self-reported symptoms following the training course (12). This indicates that some of the participants experienced a measurable degree of brain perturbation as a result of the low-level blast exposure.

The types of exposure to ballistic movement of the brain within the skull investigated in the studies mentioned above are interesting and could also be described as subconcussion (10–12). However, the current definitions suggest that head contact is the cause. We believe our new term and definition takes this into account.

## Discussion

Based on the broad inclusion criteria of these definitions, subconcussion refers to all head impacts that are below the threshold of concussion. However, in our opinion, these definitions fall short as they fail to adequately address the distinction between a head impact and the potential damage that ensues, unable to appropriately discriminate the cause-and-effect relationship between a head impact and damage. Thereby, to adequately describe this phenomenon, three components must be considered: the head impact, the injury, and the absence of observable signs and symptoms.

The long-term effects of chronically repeated subconcussive impacts are increasingly reported and it appear that the negative outcomes are only associated with multiple repeated, low level head impacts and not with a single impact or even few impacts over a shorter period (4). A single impact or a few such low level impacts appear to have no long-term effects on brain health unless they are repetitive and continued for long periods (4), further perpetuating the need for clarity and consistency when describing this phenomenon in the literature. In addition, we believe that the term “subconcussion” itself is not appropriate since it suggests that a subconcussion is a milder version of a concussion. This implies that both are a similar form of injury and it is a matter of severity that differentiates the two. Subconcussion, like concussion, is usually used to describe a single event, so the phenomenon we are referring to is associated with multiple repeated events with long term consequences. So, we believe a new term to describe the incidences and effects of chronic events is useful and appropriate.

In a recent study from our group, we reported ventricular enlargement and blood brain barrier (BBB) disruption after 60 subconcussive impacts over 2 weeks with no disruption to neuronal function (13). Parenchymal perivascular extravasation of plasma IgG was quantified to assess integrity of BBB integrity in the cortex and the hippocampal formation in these rats which exhibited significantly exaggerated cortical perivascular IgG extravasation and significant leakage of parenchymal IgG in the hippocampal region compared with the rats in the sham group (13). Furthermore, the “silent” cerebro-structural and -vascular alterations were observed not only at the impact site (cortex), but also in the deeper regions of the brain; the hippocampus and lateral ventricle, indicating a potentially distinct neuropathological pathway from those ordinarily observed in concussion. The findings from this study are not effectively explained by the many current definitions of subconcussion, which do not truly define the etiology and observed changes in brain function caused by such repeated low intensity head impacts.

The lack of precision when describing subconcussion has clinical and diagnostic implications. Given that head injury testing is not always carried out by a medical professional on the field, important symptoms may be missed if they are minor or resolve quickly. Since some concussion events may be difficult to diagnose, a mild concussion may be dismissed as a subconcussion, increasing the risk of subsequent injury if the athlete returns to play prematurely. A recent article made the distinction between concussion and mild traumatic brain injury with the following definitions. Concussion: “a complex pathophysiologic process induced by biomechanical forces, which typically results in the rapid onset of transient neurologic dysfunction that resolves spontaneously over a variable period of time and may not involve loss of consciousness” (14). Mild traumatic brain injury: “a physiologic disruption of brain function due to a traumatic injury as manifested by at least one of the following: any period of loss of consciousness, any loss of memory regarding the events immediately before or after the injury, alteration of mental state at the time of the injury or focal neurologic deficit that may or may not be transient, but without loss of consciousness >30 min, Glasgow Coma Scale of >13 beyond 30 min from the time of injury and/or post-traumatic amnesia >24 h” (14). Therefore, in order to make subconcussion distinct from concussion and mild traumatic brain injury we would like to propose a new, universal definition and name for the phenomenon.

We propose that the phenomenon be defined as “*Sudden or ballistic low magnitude movement of the brain that disrupts neurophysiological function and connectivity resulting in asymptomatic changes, with the potential to affect neural pathway function if performed repeatedly for long periods.”* The suggested term for this is “Mechanically Induced Neurophysiological Disruption” or “MIND.”

This new definition provides clarity of the phenomenon as, unlike previous definitions of subconcussion, it includes a mechanism for the injury, “…sudden and ballistic movement of the brain…,” as well as a brief description of outcomes resulting from such movements, “…asymptomatic changes, with the potential to affect neural pathway function…” and the important contributing factor of repetition of events, “…if performed repeatedly for long periods.” This definition, therefore, encompasses subconcussion, which refers to the impact, outcomes of such impacts and the important distinguishing feature of long-term repetition which can lead to some anatomical and physiological modulation of brain tissue.

## Author Contributions

APL: conception. APL, JG, and RT: organization, editing, and review of the manuscript. APL and RT: writing of the manuscript. JG and RT: critique of the manuscript. All authors contributed to the article and approved the submitted version.

## Funding

This work was supported by a grant from Neurotrauma Research Program, Perron Institute ID: DoH20193370.

## Conflict of Interest

The authors declare that the research was conducted in the absence of any commercial or financial relationships that could be construed as a potential conflict of interest.

## Publisher's Note

All claims expressed in this article are solely those of the authors and do not necessarily represent those of their affiliated organizations, or those of the publisher, the editors and the reviewers. Any product that may be evaluated in this article, or claim that may be made by its manufacturer, is not guaranteed or endorsed by the publisher.
